# Acute Encephalitis Lethargica

**Published:** 1920-04-03

**Authors:** 


### Acute Encephalitis Lethargica.

In view of the importance of early detection of cases as they arise, we are asked by the Scottish Board of Health again to bring some of the diagnostic points to the notice of all concerned in health matters.

The essential characters of the malady are those of an acute general disease with predominant indications of cerebral affection, progressive langour, apathy and drowsiness, passing into complete lethargy, muscular weakness passing into complete disablement, and various paralyses of muscles chiefly of the eyes and face. It is considered by analogy to be an infectious disease different from any hitherto recognised. Its nearest clinical affinity is to that form of acute poliomyelitis which affects the brain rather than the medulla (acute polio-encephalitis); but it is to be distinguished therefrom by a supervention of progressive stupor, a feature which in acute polioencephalitis is notably absent. The localised limb paralysis that is a distinctive feature of acute poliomyelitis has not been observed in acute encephalitis lethargica.

There are epidemic as well as clinical distinctions between acute poliomyelitis and acute encephalitis lethargica; for instance, acute encephalitis lethargica is most frequent in childhood, gradually diminishing in incidence up to the age of sixty; acute poliomyelitis is far more common in infancy than in later life, and is rare over forty. Again, acute encephalitis lethargica is most fatal in middle life (age period thirty to forty); acute poliomyelitis is vastly more fatal in childhood and youth than in later years.

Acute encephalitis lethargica, though it has occurred in England in epidemic form, has never attained to extensive proportions. But it is important that in all parts of Great Britain an informed outlook should be kept for its recurrence. Little is known regarding its essential nature, the channels by which it is spread or, consequently, the means by which it may be prevented and controlled. It is, therefore, desirable that cases suspected to be of the nature of acute encephalitis lethargica or even considered remotely to resemble it, should be intimated to the Medical Officer of Health, who should afford every facility towards confirmatory diagnosis, treatment, and such isolation as he considers necessary. With this end in view, it is desirable also to bring to his notice cases of ordinary acute poliomyelitis.

All that had been ascertained concerning this disease eighteen months ago is to be found in the "Report to the Local Government Board (England) of an Enquiry into an Obscure Disease, Encephalitis Lethargica," 1918, published by H.M. Stationery Office, price 2s. 6d.

